# Dynamic Sounds Capture the Boundaries of Peripersonal Space Representation in Humans

**DOI:** 10.1371/journal.pone.0044306

**Published:** 2012-09-28

**Authors:** Elisa Canzoneri, Elisa Magosso, Andrea Serino

**Affiliations:** 1 Dipartimento di Psicologia, ALMA MATER STUDIORUM - Università di Bologna, Bologna, Italy; 2 Centro studi e ricerche in Neuroscienze Cognitive, Polo Scientifico-Didattico di Cesena, Cesena, Italy; 3 Dipartimento di Elettronica, Informatica e Sistemistica, ALMA MATER STUDIORUM - Università di Bologna, Bologna, Italy; Macquarie University, Australia

## Abstract

**Background:**

We physically interact with external stimuli when they occur within a limited space immediately surrounding the body, i.e., Peripersonal Space (PPS). In the primate brain, specific fronto-parietal areas are responsible for the multisensory representation of PPS, by integrating tactile, visual and auditory information occurring on and near the body. Dynamic stimuli are particularly relevant for PPS representation, as they might refer to potential harms approaching the body. However, behavioural tasks for studying PPS representation with moving stimuli are lacking. Here we propose a new dynamic audio-tactile interaction task in order to assess the extension of PPS in a more functionally and ecologically valid condition.

**Methodology/Principal Findings:**

Participants vocally responded to a tactile stimulus administered at the hand at different delays from the onset of task-irrelevant dynamic sounds which gave the impression of a sound source either approaching or receding from the subject’s hand. Results showed that a moving auditory stimulus speeded up the processing of a tactile stimulus at the hand as long as it was perceived at a limited distance from the hand, that is within the boundaries of PPS representation. The audio-tactile interaction effect was stronger when sounds were approaching compared to when sounds were receding.

**Conclusion/Significance:**

This study provides a new method to dynamically assess PPS representation: The function describing the relationship between tactile processing and the position of sounds in space can be used to estimate the location of PPS boundaries, along a spatial continuum between far and near space, in a valuable and ecologically significant way.

## Introduction

The space immediately surrounding the body, i.e. Peripersonal Space (PPS) [1]–[3], mediates every physical interaction between the body and the external world, because it is within its boundaries that we can reach and act upon objects, as well as avoid looming threats. The primate brain contains a specific network of neural populations coding the location of nearby stimuli in relation to different parts of the body. In monkeys, multisensory neurons in fronto-parietal areas in the ventral premotor cortex (vPM; F4, [4] or polysensory zone PZ, [5]–[8]), in the ventral intraparietal area on the fundus of the intraparietal sulcus (VIP; [9]–[11]), in the parietal areas 7b, and in the putamen [12] integrate somatosensory information with visual and acoustical information within PPS. These neurons respond both to tactile stimuli on the monkey’s arm, face or torso, and to visual and/or acoustic stimuli presented close, but not far (i.e. at more than 30 cm) from the same body part (see [2] for a review).

The existence of a similar system in the human brain has been initially supported by neuropsychological studies on right-brain damaged patients suffering from cross-modal extinction. In these patients, the perception of a contralesional tactile stimulus is strongly affected by concurrently presenting an ipsilesional visual, or auditory stimulus, close to the patient’s hand [13], [14] or face [15], [16] but perception is less importantly modulated when stimuli are presented at a distance (i.e. in ‘far’ or ‘extrapersonal’ space) [13], [16], [17]. The study of crossmodal extinction has brought a considerable contribution to our understanding of how the integration of stimuli perceived in multiple sensory modalities is used by the brain to build coherent representations of the space directly surrounding the human body (see [18] for a review). In healthy subjects, investigations into the interactions between visual/auditory and tactile events have revealed spatial constraints of multisensory interaction. Behavioural studies using visual-tactile interaction paradigms (Crossmodal Congruency Task, [19], [20]) or audio-tactile interaction paradigms (Temporal Order Judgment task, [21]; Redundant signals effects, [22]; audio-tactile interaction paradigm, [23]–[25]; audio-tactile Crossmodal Congruency Effect study, [26]) confirmed that these forms of multisensory integration are stronger when stimuli occur near the body. These findings suggest that PPS representation is enabled through the integration of somatosensory information related to specific body parts and visual and/or acoustic information related to objects presented in a limited portion of space surrounding the same body parts (see [27] for a review). Finally, neuroimaging studies using fMRI [28]–[31], TMS [25] and EEG [32] demonstrated that multisensory representation of PPS in the human brain is implemented in premotor and posterior parietal areas largely corresponding to vPM and VIP areas in the monkey brain.

The vast majority of the previously cited behavioural studies on PPS representation compared the effects of visual or auditory stimuli, presented at two fixed locations – far or close to the body - on tactile perception. Interestingly, neural systems representing PPS both in humans and in monkeys show response preference for moving stimuli, over static stimuli. Indeed, neurophysiological studies in monkeys showed that bimodal and trimodal neurons, both in the premotor cortex [5]–[8] and in the ventral intraparietal area [9], [33], are more effectively activated by presenting three dimensional objects approaching toward and receding from the animal’s body, compared to static stimuli. Some of these neurons also show direction-selective and velocity dependent response patterns, as firing rates in certain cells increase as a function of the velocity of approaching stimuli [6]. In humans, Bremmer and colleagues [28] demonstrated an increased neural activity in the depth of the intraparietal sulcus and in the ventral premotor cortex evoked by approaching visual, auditory and tactile stimuli (see also [29]).

The high sensitivity of the PPS system for dynamic stimuli fits well with the sensory-to-motor function of PPS representation. Such representation codes for the spatial position and dynamics of an external stimulus with respect to a part of the body potentially interacting with it in order to plan defensive reactions to potential looming threats [2] or approaching movements toward interesting objects [1].

Given the high relevance of moving objects to the PPS system, we propose that using dynamic, instead of static stimuli could be a more powerful way to study PPS representation in humans. Moreover, this approach more directly resembles ecological contexts, where external stimuli continuously move in the environment. Finally, this approach is closer to the experimental conditions used in monkeys’ neurophysiology, thus allowing a more direct comparison across species. For these reasons, in the present study we present a new paradigm, which involves carrying out a dynamic audio-tactile interaction task in order to assess the extension of PPS in a more functionally and ecologically valid condition. We measured reaction time (RTs) to a tactile stimulus at the hand while dynamic sounds were presented, giving the impression of a sound source either approaching, or receding from the subject’s hand. Tactile stimulation was delivered at different temporal delays from the onset of the sound, such that it occurred when the sound source was perceived at varying distances from the body. Subjects were asked to respond as rapidly as possible to the tactile stimulation, trying to ignore the sound. The rationale of the task is that stimuli from different sensory modalities interact more effectively with one another when presented within the same spatial representation [34]. Since we have repeatedly demonstrated that sounds boost tactile RTs when presented close to the body, and not at a distance [23]–[25], we predicted that RTs to tactile stimuli would progressively decrease as a function of the sound source’s perceived approach; and conversely, that they would increase as a function of the sound source’s perceived recession. The function describing the relationship between tactile RTs and the perceived position of sounds in space at the occurrence of the tactile stimulation can be used to study the shape of PPS representation and to locate its boundaries along a continuum between near and far space.

## Methods

### Subjects

Seventeen healthy subjects (16 females, mean age 23.2 years, range: 20–26) participated in the study. All participants were right-handed and had normal hearing and touch. All subjects (students at the University of Bologna) gave their written informed consent to participate in the study, which was approved by the Ethical Committee of Department of Psychology, University of Bologna, and was performed in accordance with the Declaration of Helsinki.

### Procedures and Materials

During the audio-tactile interaction task, subjects were blindfolded and sat down with their right arm resting palm down on a table beside them. On each trial, a sound (pink noise) was presented for 3000 ms. Two types of sound were used, which we term from here onwards as *IN* and *OUT* sounds. The sounds were generated by two loudspeakers: one was placed on the table in the proximity of the hand, while the other one was placed on the floor, at a distance of ∼100 cm from the near loudspeaker (i.e. far from the hand). Auditory stimuli were samples of pink-noise, at 44.1 kHz. Sound intensity was manipulated by using the SOUNDFORGE 4.5 software (Sonic Foundry, Madison, WI), so that IN sounds had exponentially rising acoustic intensity from 55 to 70 dB Sound Pressure Level (SPL) as measured with an audiometer at the position of subjects’ ears, while OUT sounds had exponentially falling acoustic intensity from 70 to 55 dB. Each sound is a combination of two identical samples of pink noise, one of increasing (for the IN sound) and the other one of decreasing (for the OUT sound) intensity, emitted by the near and the far loudspeaker. Both loudspeakers were activated simultaneously, but in case of the IN sound the far loudspeaker activated at the maximum intensity and then its intensity decreased up to silence along the trial, whereas the near loudspeaker activated at the minimum intensity (not perceived), and then its intensity increased up to the maximum value along the trial. In order to generate the OUT sound, the same setting was used, with reversed intensities and timing for the near and far loudspeaker. In this way, IN sounds gave the impression of a sound source moving from the far to the near loudspeaker, i.e., towards the subject’s body, while OUT sounds gave the impression of a sound source moving in the opposite direction. Although other cues, such as frequency spectrum, reverberant energy and inter-aural level differences, are normally used by the auditory system to determine the spatial position of a sound, dynamic change in sound intensity seems to provide the most critical information for determining the position and direction of a moving auditory source [35], [36].

Along with the auditory stimulation, in the 60% of trials subjects were also presented with a tactile stimulus, delivered by means of a constant-current electrical stimulator (DS7A, Digitimer, Hertfordshire, United Kingdom), via a pair of neurological electrodes (Neuroline, Ambu, Ballerup, Denmark) placed on the hairy surface of the right index. The electrical stimulus was a single, constant voltage, rectangular monophasic pulse. Before the experiment, the intensity of the tactile stimulus was set to be clearly above thresholds, individually for each subject, as follows: intensity of the stimulator was set at the minimum value and then progressively increased until the subject referred to clear perceive the stimulation. Then, the subject was presented with a series of 10 stimuli, at that level of stimulation, intermingled with 5 catch trials, and asked to report when he/she felt the tactile stimulus. If the subject did not perfectly perform (i.e., if he/she omitted some stimuli or answered to catch trials), intensity was further increased by 5 mA, and the procedure was repeated. Intensity for the tested subjects ranged between 60 and 90 mA, depending on subjects’ individual thresholds. Stimulus duration was equal to 100 µsec. Along the experiment, the remaining trials (40% out of total) were catch trials with auditory stimulation only. Subjects were asked to respond vocally to the tactile target, when present, saying “TAH” as fast as possible, trying to ignore the auditory stimulus. Tactile RTs were recorded by means of a voice-activated relay. A PC running C.I.R.O. software (www.cnc.unibo.psice.unibo/ciro) was used to control the presentation of the stimuli and to record responses.

For each trial, the sound was preceded and followed by 1000 ms of silence. Temporal delays for the tactile stimulus were set as follows: *T1*, tactile stimulation administered at 300 ms after the sound onset (corresponding to 1300 ms from the beginning of the trial); *T2*, at 800 ms from sound onset (at 1800 ms from trial beginning); *T3*, at 1500 ms from sound onset (at 2500 ms from trial beginning); *T4*, at 2200 ms from sound onset (at 3200 ms from trial beginning); and *T5*, at 2700 ms from sound onset (at 3700 ms from trial beginning). Thus, the critical manipulation was that the tactile stimulus was delivered at different temporal delays (from T1 to T5) from the onset of the auditory stimulus, for both IN and OUT sounds. In this way, tactile stimulation occurred when the sound source was perceived at different locations with respect to the body: i.e., close to the body, at high temporal delays for the IN sound and at low temporal delays for the OUT sound; and far from the body, at low temporal delays for the IN sound and at high temporal delays for the OUT sound.

**Figure 1:**
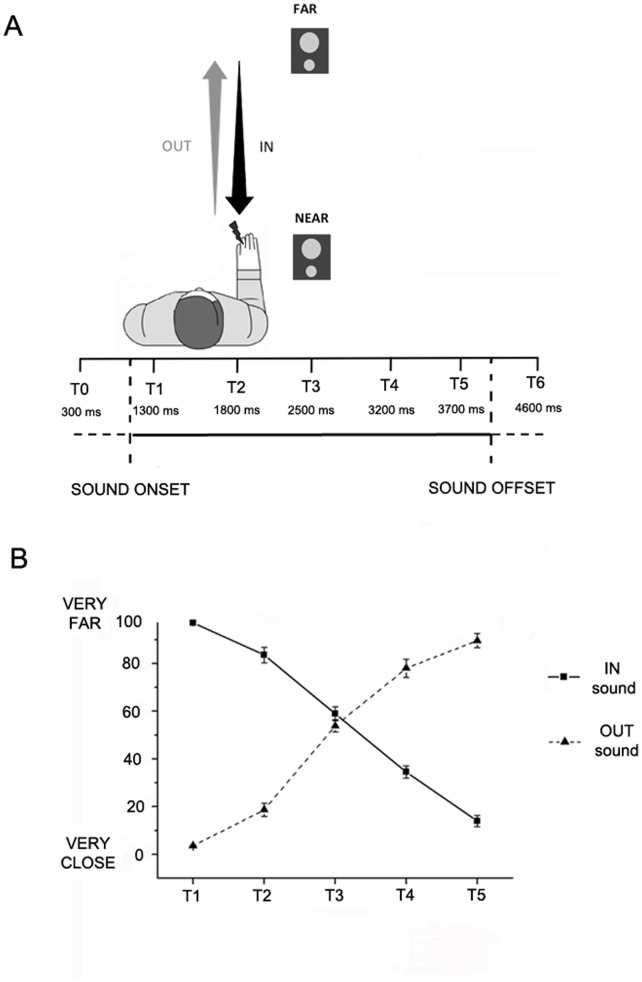
Experimental setup. Panel A. Procedure. Subjects received a tactile stimulus at their hand while task-irrelevant sounds either approached to or receded from the hand. Tactile stimuli were delivered at different temporal delays from sound onset (from T1 to T5), so that they were processed when sounds were perceived at a different distance from the hand. Panel B. *S*ound localization experiment results. The graph shows subject’s mean responses indicating the perceived position of sound in space when they receive a tactile stimulus at different temporal delays from sound onset, from T1 to T5. Filled line refers to IN sound condition, hatched line refers to OUT sound condition. Error bars denote S.E.M. A repeated measure ANOVA with Sound (IN, OUT) and Temporal Delay (from T1 to T5) confirmed that IN and OUT were perceived as an approaching and receding auditory stimuli, respectively, as clearly shown by the significant two-way interaction (F(4,24) = 304.30, p<0.00001).

Finally, in order to measure RTs in unimodal tactile condition (without any sound), tactile stimulation could be also delivered during the silence periods, preceding or following sound administration, namely at 300 ms (*T0*) and at 4600 ms (*T6*) after the beginning of the trial (see Figure 1A). The total experiment consisted in a random combination of 8 target stimuli for each temporal delay, for the IN and OUT sounds, resulting in a total of 112 trials with a tactile target, randomly intermingled with 76 catch trials. Trials were equally divided in two blocks, lasting about 8 minutes each.

In order to demonstrate that subjects actually perceived the sound source at different locations according to different temporal delays (from T1 to T5) for the IN and the OUT sound, we ran a sound localization experiment on 7 naïve subjects. During the sound localization experiment subjects were blindfolded and sat down with their right arm resting palm down on a table beside them. They received a tactile stimulation on the forearm at one of the different temporal delays in a series of 80 trials, randomly presented. At the end of each trial, they were asked to verbally indicate the perceived position of the sound in space when they had felt the tactile stimulus, on a scale from 1 (very close) to 100 (very far). Participants were explicitly invited to use the entire range between 1 and 100, taking in account also for small differences in the perceived position of sound. Subjects’ responses and statistical analyses, reported in Figure 1B, clearly show that for the IN sound, subjects progressively perceived the sound closer to their body when the tactile stimulus was administered at successive temporal delays from T1 to T5. The pattern of responses was reversed for the OUT sound, when the sound was perceived in spatial positions progressively farther from the body from T1 to T5. The results of this control experiment confirmed that IN and OUT sounds were perceived respectively as approaching and receding auditory stimuli, and that, when subjects received tactile stimulation at different temporal delays, the sound was perceived at a different distance from their body.

## Results

**Figure 2:**
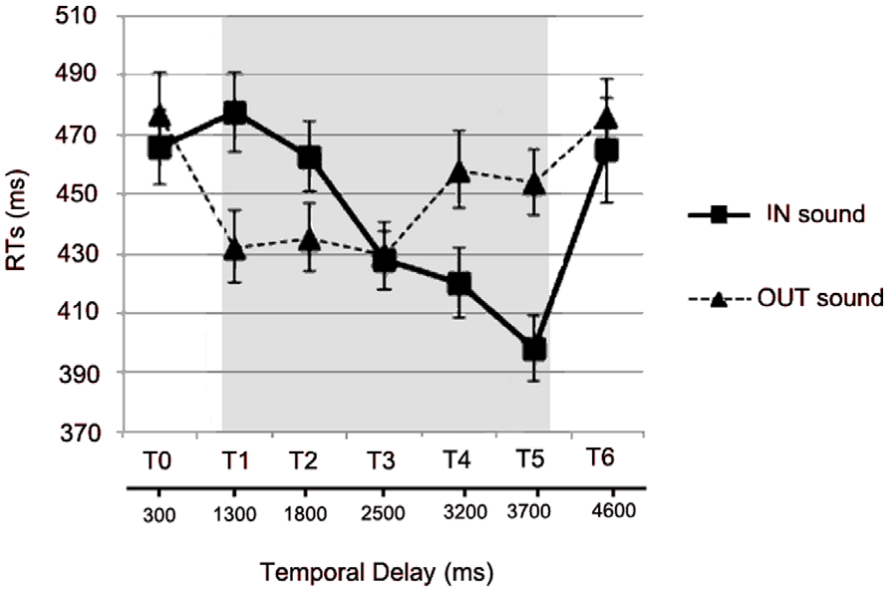
Effects of IN and OUT sounds on tactile processing. Mean RTs (and S.E.M.) to the tactile target at different temporal delays (from T0 to T6) for IN (filled line) and OUT (hatched line) sounds. The shaded region indicates the duration of the sounds.

Since tactile stimuli were administered well above threshold, subjects were extremely accurate in performing the task, as rate of false alarms and omissions was very low, i.e., 0.25% and 1.75% respectively. Thus, the performance was analysed in term of reaction time only. Mean RTs to tactile targets were calculated for every temporal delay, from T0 to T6, separately for IN and OUT sounds. RTs exceeding more than 2 standard deviations from the mean RT were considered outliers and trimmed from the analyses (1.6% of trials on average in all conditions). The relationship between RTs to the tactile target and the different temporal delays at which the tactile stimulus was administered (from T0 to T6) is represented in Figure 2 for the IN (filled line) and the OUT (hatched line) sound. Two different effects are visible: for the IN sound, RTs progressively decreased as temporal delays increased, i.e. as the perceived sound approached the body; and vice versa for the OUT sound: RTs progressively increased as temporal delays increased, i.e. as the perceived sound receded from the body. However, the shape of the relationship between RTs and Sound position does not appear exactly the same for the two classes of sounds.

These effects were confirmed by an ANOVA on tactile RTs with the within subjects factors of Sound (IN, OUT) and Temporal Delay (T0, T1, T2, T3, T4, T5, T6). The two-way interaction Sound x Temporal Delay was significant (F(6,96) = 7.88; p<0.0001). In order to analyse this interaction, we performed two separate ANOVAs for IN and OUT sounds with Temporal Delay as within-subjects factor. In case of the IN sound, the ANOVA revealed a significant main effect of Temporal Delay (F(6,96) = 8.46, p<0.0001). Newman-Keuls post-hoc tests showed that RTs at T1 (when sounds were perceived far from the body; mean RTs = 478 ms, S.E.M. = ±18) and T2 (463 ms±15) were significantly slower compared to RTs at T3 (when sounds were perceived close to the body; 428 ms±14), T4 (420 ms±15) and T5 (398 ms±15; all p_s_<0.05). RTs at T1 and T2 were not significantly different from each other (p = 0.73), as well RTs at T3, T4 and T5 were not significantly different from each other (all p_s_>0.10). RTs in the two unimodal conditions (i.e., when tactile stimuli were delivered at T0, before sound onset and at T6, after sound offset) were significantly slower as compared to RTs at T3, T4 and T5 (all p_s_<0.05), i.e. when the sound was perceived close to the body. In addition, RTs at T0 and T6 were not different from each other (RTs at T0 = 467 ms±17 and T6 = 465 ms±23, p = 0.95), therefore excluding the possibility that subjects were generically faster at late delays in each trial just because they paid more attention as the probability of receiving a stimulation increased along the trial duration.

In the case of the OUT sound, the main effect of Temporal Delay was significant (F(6,96) = 5.97, p<0.0001), as it was for the IN sound. RTs at T5 (454 ms±15), and T4 (458 ms±17) - when the sound was perceived far from the body - were slower than RTs at T3 (429 ms±15), T2 (435 ms±15) and T1 (432 ms±16) - when sounds were perceived close to the body;. The pattern of results therefore showed a similar trend as for the IN sound. However, the differences between RTs at higher temporal delays (T5 and T4) and RTs at lower temporal delays (T1–T3) were statistically significant with simple comparisons (two-tailed t-tests, p_s_<0.05), but did not resist to Newman-Keuls corrections for multiple comparisons (all p_s_>0.16). RTs in the two unimodal conditions were not different from each other (RTs at T0 = 477 ms±18 and T6 = 476 ms±17, p = 0.55), but were significantly slower as compared to the other conditions (all ps <0.05, Newman-Keuls corrected).

Taken together, these results suggest that tactile processing is modulated by co-occurrence of dynamic sounds, depending on the position of sounds in space, as far as sounds were perceived at a limited distance from the body, and such distance can be considered as the boundary of PPS representation around the hand. In addition, the relationship between the spatial position of sounds in space and their effect on tactile RTs seems stronger when an approaching, rather than when a receding sound, was presented.

**Figure 3:**
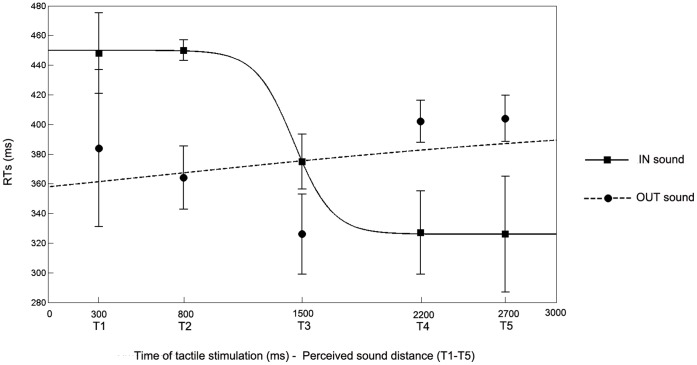
Best fitting function for the relationship between sound position in space and tactile processing. Data from a paradigmatic subject are reported. Figure 3 plots mean RTs (and S.E.M.) at different times of tactile stimulus delivery and the best fitting sigmoidal functions for IN (filled line) and OUT (hatched line) sounds.

In order to further investigate the differential effects of the two types of dynamic sounds on tactile processing, we studied mathematical functions describing the relationship between tactile RTs and timing at which tactile stimuli were delivered. We compared two possible functions, a sigmoidal function and a linear function. In order to compute the mathematical functions, the time of tactile stimulation was referred to the sound onset for both types of sounds (IN and OUT sounds), so that experimental time T1 corresponds to 300 ms, T2 to 800 ms, T3 to 1500 ms, T4 to 2200 ms and T5 to 2700 ms. The sigmoidal function was described by the following equation: 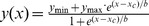 where *x* represents the independent variable (i.e., the timing of touch delivery in ms), *y* the dependent variable (i.e., the reaction time), *y_min_* and *y_max_* the lower and upper saturation levels of the sigmoid, *x_c_* the value of the abscissa at the central point of the sigmoid (i.e., the value of *x* at which *y* = (*y_min_*+ *y_max_*)/2) and *b* establishes the slope of the sigmoid at the central point. The linear function was described by the following equation: y(x) = y_0_+ k · x; where *x* and *y* have the same meaning as above, *y_0_* represents the intercept at *x* = 0 and *k* is the slope of the linear function. For each subject, the two functions were fitted to the averaged tactile RTs at the five timing of tactile delivery, separately for the IN and the OUT sound, in the least-squares sense. In the linear model, the estimated parameters were the intercept (*y_0_*) and the slope (*k*). In the sigmoidal model, we analogously limited the estimated parameters to two, as in the linear function, in order to directly compare the root mean square error (RMSE), as an index of best fit between the two models. To this end, for each set of data, values of the parameters *y_min_* and *y_max_* were assigned a priori equal to the minimum and maximum values of the data set (independently calculated for each subject), and the estimated parameters were the central position of the sigmoid (*x_c_*) and the slope of the sigmoid at the central point (*b*). For the IN sound, RMSE was significantly lower for the sigmoidal function (19.60 ms) than for the linear function (22.54 ms; t(16) = −2.43, p<0.05), indicating that the empirical data were better represented by the former than by the latter function (see Table 1). This finding suggests that the effect of IN sounds on tactile processing did not increase linearly along a continuum from far to near space as the sound approaches the body. Instead, there was a critical spatial range, located between T2 and T3, after which auditory stimuli from the outside began interacting with tactile stimuli on the body surface, fastening tactile RTs. We can consider such spatial range as the boundary of audio-tactile PPS representation around the hand (see Figure 3).

**Table 1:** Estimated parameters and Root Mean Square Errors for the sigmoidal function (central position and slope, on the left) and the linear function (intercept and slope, on the right) fitting the relationship between tactile RTs and timing of touch delivery −300 ms, 800 ms, 1500 ms, 2200 ms, 2700 ms, corresponding to the five different perceived positions of the sounds - both for IN and OUT sound.

	SIGMOIDAL FUNCTION			LINEAR FUNCTION		
	CENTRAL POSITION (ms)	SLOPE	RMSE (ms)	INTERCEPT (ms)	SLOPE	RMSE (ms)
**IN**	1439.22	−0.15	19.60	486	−0.03	22.54
**OUT**	1425.02	0.03	20.59	426	0.02	20.70

In the case of the OUT sound the function linking tactile RTs and the perceived position of sounds in space did not fit the data as well as that for the IN sound. Indeed, RMSE for the sigmoidal function (20.59 ms) were not significantly different from those for the linear function (20.70 ms; t(16) = −0.19; p = 0.85). Moreover, the slope of the sigmoidal function computed for the OUT sound was significantly flatter (0.03 ms) compared to that computed for the IN sound (−.15 ms; p<0.05). These results suggest that the effect of sound on tactile RTs depends not only on the perceived position of sound in space, but also on the perceived direction of sound motion, with a stronger effect for IN sounds than for OUT sounds.

## Discussion

In this study, we developed a new dynamic paradigm to study PPS representation. Our results show that an auditory stimulus speeds up the processing of a tactile stimulus at the hand, if the sound is administered within a limited distance from the hand. By using dynamic sounds we were able to study such critical distance along a continuous spatial range, spanning near and far space, thus estimating the boundaries of PPS representation.

Previous studies have shown that auditory stimuli affect the perception of tactile stimuli, both in terms of detection ability [37] and RTs [21], [23], [24]. However, whether, and to what extent audio-tactile interactions are modulated by the spatial features of the stimuli is still a debated issue. Some studies [21], [38]–[40] have suggested that the spatial links between auditory and tactile signals may be weaker than those existing between other modality pairings involving vision, such as audio-visual [41] and visuo-tactile [19], [42] interactions. Indeed, some authors reported a facilitation effect not only when auditory and tactile stimuli are delivered to the same location, but also when they are widely separated ([21], [39]; see also [43], [44]). In contrast, other studies have supported the hypothesis that spatial factors, such as the stimulus distance from the body, are also important in auditory-tactile interactions, as they showed stronger auditory–tactile effects for stimuli arising from the same sector of space [22], [45], [46].

The auditory system has a lower spatial acuity than the visual system, thus the modulation of tactile processing in relation to auditory stimuli might be less sensitive to spatial factors, compared with visual stimulation. However, if spatial features of auditory stimulation are stressed by using dynamic sounds, as in the present experiment, spatially dependent auditory-tactile interactions can be revealed. It is worth also noting that most previous evidence of space-dependent modulation of auditory-tactile interactions in monkeys [8], [11] concerns stimuli administered close to the head, and especially in the rear space. Also in humans, evidence concerning spatially dependent audio-tactile interactions is more common for the peri-head space than in the peri-hand space (see [47] for a review), although a number of studies reported different forms of audio-tactile interaction around the hand [48], [49]. This might have occurred because localization of auditory stimuli with respect to the head is simpler and more precise than that with respect to the hand, due to the nature of computation required to localize sounds in space. Nevertheless, localization of sounds around the hand is necessary under specific conditions. For instance, when you hear a bee approaching your right hand you do not withdraw your head or your left hand, but you do withdraw your right hand. In cases such as this, auditory-tactile interaction in the peri-hand space is likely to be modulated as a function of the position of sounds in space, as we demonstrated in the present study. Accordingly, previous research from our group demonstrated that the detection of a tactile stimulus delivered on the hand is accelerated if a concurrent sound is presented near the hand rather than far away [23], [24]. Critically, this effect vanished when subjects retracted their hand from the source of the near sound (while keeping constant the distance from the head) [25], showing that these audio–tactile interaction effects are sensitive to the position of the hand in space, or, more generally, they are coded in a reference frame centred on the body part where tactile stimuli are administered. In future studies it might be interesting to study the effect of other possible sound trajectories on PPS representation. For instance it is well know that sound localization is more precise for side-to-side trajectories than for frontal trajectory, because in the former case, cues based on interaural differences are stronger [36]. So, it is possible that, using this paradigm, the function describing the relationship between tactile RTs and sound positions is more sensitive along side-to-side trajectories that along the frontal direction. It is also possible that the critical position in space where sounds begin affecting tactile RTs is localized at a different distance from the body for side-to-side as compared to frontal directions. Indeed, PPS could differently extend in the front space where both hands can immediately and co-ordinately act, as compared to the space aside the body, where bimanual actions do not occur. It is worth noting, however, that in the everyday life we are more likely to interact with stimuli presented in the frontal space. For this reason, in this first study we decided to test audio-tactile interactions on a frontal plane.

We proposed that the speeding effect on RTs due to sounds processed in the near space might arise from the integration of multisensory (auditory and tactile) inputs within the same spatial representation (i.e. within PPS around the hand). Similarly, inhibitory r-TMS over multisensory areas in ventral premotor cortex and in posterior parietal cortex abolish the speeding effect due to near sounds [25]. A similar mechanism can explain results from the present study.

The novelty of the present approach is that by using dynamic stimuli, approaching or receding from the body, we could measure multisensory interaction around the body along a continuum between far and near space, rather than as a comparison between a series of fixed locations. This approach offers a series of advantages in comparison to previous behavioural approaches, which compared the effects on tactile processing of visual [27] or auditory [47] stimuli presented in two fixed locations. First, we measured the extension of PPS in a more ecologically valid condition, mimicking dynamic stimulations of everyday life. Second, this paradigm directly resembles the stimulations used in monkey neurophysiology to study PPS bimodal or trimodal neurons, where a visual or an auditory stimulus was presented, as approaching to or receding from the animal’s body part where the neuron’s tactile receptive field was located [6], [8], [9], [11]. Our approach also fits well with the notion that bimodal and trimodal neurons in monkeys’ premotor [5]–[8] and parietal cortices [9], [33] and multisensory responses in human homologues areas [28], [29] are particularly sensitive to dynamic stimuli.

Interestingly, the present results also suggest that, among dynamic stimuli, approaching sounds have a stronger spatially-dependent effect on tactile processing, compared with receding sounds. Indeed, the sigmoidal function, describing the relationship between tactile RTs and timing at which tactile stimuli were delivered, had a better fit and was significantly steeper for the IN sound than for the OUT sound. These results are in keeping with several studies showing that primates sensory systems are particularly sensitive to approaching stimuli. Indeed, an attentional bias toward approaching stimuli was shown in monkeys, both in visual and auditory domains, as compared with receding stimuli [50]–[52]. In addition, bimodal and/or trimodal neurons in multisensory brain areas in the ventral premotor cortex and in the posterior parietal cortex in monkeys were shown to respond preferentially to approaching visual [9], [33], [53] and auditory [8], [50] stimuli as compared to receding stimuli. At a behavioural level, human subjects show a perceptual bias to detect an approaching, rather than a receding, stimulus [54]–[56]. Human listeners also underestimate time of contact with the body of an approaching sound as compared to a receding sound [35], [57]. Moreover, audio-visual integration is stronger for approaching than for static or receding stimuli [54], [56], [58]. Thus, approaching stimuli have been shown to be particularly relevant at different levels of information processing [59]. The present study provides a further level of evidence for this argument by showing a stronger effect of approaching sounds in modulating tactile processing.

These findings fit perfectly with the sensory-motor function of PPS representation [2]: a stimulus possibly colliding with the body implies faster and more accurate processing, as it is likely to require a rapid motor response, in order to avoid a potential harm. This notion is supported by electrophysiological evidence in monkeys showing that direct electrical stimulation of the areas containing bimodal and trimodal PPS neurons evokes in anesthetized animals fast motor responses, resembling defensive and avoidance reactions to threatening stimuli in everyday life contexts [60]–[62]. The existence of a similar sensory-to-motor coding of PPS in humans is supported by two recent TMS studies showing that processing visual [63] or auditory [64] stimuli within or outside the PPS around the hand differently affects the representation of hand muscles in the cortico-spinal tract.

In sum, the present study provides an effective and ecologically valid approach to measure the extent of PPS representation. The function describing the relationship between tactile processing and the position of sounds in space along a spatial continuum can be used to localize the boundaries of PPS representation. We show that such a relationship is better described by a sigmoidal (rather than linear) function, meaning that RTs sharply decreased as a sound crosses a spatial limit, past which the addition of an auditory stimulus speeds up the detection of a tactile stimulus administered on the body. Such spatial limit can be considered as the boundary of PPS representation. This method can be used to study plasticity of PPS representation in different contexts and following different types of short term and long term experiences [65].
